# Identification and expression analysis of microRNAs and targets in the biofuel crop sugarcane

**DOI:** 10.1186/1471-2229-10-260

**Published:** 2010-11-24

**Authors:** Almir S Zanca, Renato Vicentini, Fausto A Ortiz-Morea, Luiz EV Del Bem, Marcio J da Silva, Michel Vincentz, Fabio TS Nogueira

**Affiliations:** 1 Centro de Biologia Molecular e Engenharia Genetica (CBMEG), Universidade Estadual de Campinas, Campinas, SP, Brazil; 2 Centro de Biotecnologia Agricola (CEBTEC), Escola Superior de Agricultura "Luiz de Queiroz", (ESALQ)/USP, Piracicaba, SP, Brazil; 3 Depto de Biologia Vegetal, Instituto de Biologia, UNICAMP, Campinas, SP, Brazil; 4 Depto de Genetica, Instituto de Biociencias, Universidade Estadual Paulista (UNESP), Botucatu, SP, Brazil

## Abstract

**Background:**

MicroRNAs (miRNAs) are small regulatory RNAs, some of which are conserved in diverse plant genomes. Therefore, computational identification and further experimental validation of miRNAs from non-model organisms is both feasible and instrumental for addressing miRNA-based gene regulation and evolution. Sugarcane (*Saccharum spp*.) is an important biofuel crop with publicly available expressed sequence tag and genomic survey sequence databases, but little is known about miRNAs and their targets in this highly polyploid species.

**Results:**

In this study, we have computationally identified 19 distinct sugarcane miRNA precursors, of which several are highly similar with their sorghum homologs at both nucleotide and secondary structure levels. The accumulation pattern of mature miRNAs varies in organs/tissues from the commercial sugarcane hybrid as well as in its corresponding founder species *S. officinarum *and *S. spontaneum*. Using sugarcane *MIR827 *as a query, we found a novel *MIR827 *precursor in the sorghum genome. Based on our computational tool, a total of 46 potential targets were identified for the 19 sugarcane miRNAs. Several targets for highly conserved miRNAs are transcription factors that play important roles in plant development. Conversely, target genes of lineage-specific miRNAs seem to play roles in diverse physiological processes, such as *SsCBP1*. *SsCBP1 *was experimentally confirmed to be a target for the monocot-specific miR528. Our findings support the notion that the regulation of *SsCBP1 *by miR528 is shared at least within graminaceous monocots, and this miRNA-based post-transcriptional regulation evolved exclusively within the monocots lineage after the divergence from eudicots.

**Conclusions:**

Using publicly available nucleotide databases, 19 sugarcane miRNA precursors and one new sorghum miRNA precursor were identified and classified into 14 families. Comparative analyses between sugarcane and sorghum suggest that these two species retain homologous miRNAs and targets in their genomes. Such conservation may help to clarify specific aspects of miRNA regulation and evolution in the polyploid sugarcane. Finally, our dataset provides a framework for future studies on sugarcane RNAi-dependent regulatory mechanisms.

## Background

MicroRNAs (miRNAs) are small regulatory RNAs (19-21 nt) that play crucial roles in diverse aspects of plant development [1-3], biotic and abiotic stress responses [4,5], signal transduction and protein degradation [6,7]. MiRNAs are generated by stepwise processing of RNA polymerase II (Pol II)-dependent primary miRNA transcripts (pri-miRNAs). The pri-miRNAs typically form an imperfect fold-back structure, which is processed into a stem-loop precursor (pre-miRNA) and further excised as an RNA duplex by the DICER-LIKE1 (DCL1) enzyme. Partial or complete base-pairing between the miRNA and its target RNA allows the miRNA-associated RNA-induced silencing complexes (RISCs) to promote translational inhibition, accelerated exonucleolytic mRNA decay, and/or mRNA cleavage through slicing within miRNA-mRNA base-pairing (for review, see [8]). The majority of the target genes of highly conserved miRNAs are transcription factors that play important roles in development [9]. Conversely, lineage-specific miRNAs seem to regulate the expression of a broader type of genes, including those involved in cellular metabolism, stress response, and post-translational modifications [7,10].

The identification of miRNAs and their targets in a large number of plant species is an important step to understand the function and evolution of miRNAs and miRNA-dependent gene regulation. Over 1,300 miRNAs from eudicotyledoneous and 832 miRNAs from monocotyledonous plants have been deposited in the latest release of miRBase (release 14.0 September 2009). Although deep sequencing methods have substantially contributed to the identification of conserved and lineage-specific miRNAs in model species [11], these approaches are time-consuming and relatively expensive. In this regard, public EST databases and genomic survey sequences (GSSs) have become attractive alternatives to identify non-coding sequences through computational approaches in non-model plants.

The fact that most known miRNAs are evolutionarily conserved raises the possibility of identifying new miRNA homologs in other species using computer-based strategies [12], and such *in silico *approaches have been reviewed and classified not only as homology-based but also as structure similarity-based searches [7,13]. Therefore, recent computational methods provide an accurate, fast, inexpensive, and consequently convenient way to retrieve miRNA precursor sequences from publicly available sequence databases. Finally, target mRNAs of conserved miRNAs can be searched using web-based [7] or in-house algorithms and analyzed across plant species.

Most identified miRNAs and their targets have been predicted in plants for which whole genome information is available such as *Arabidopsis thaliana *and rice. Currently, there is no experimental and only scarce computational information about miRNAs and their targets in sugarcane (*Saccharum spp.*). Sugarcane is an economically important biofuel crop. Recently, it has become a target for improvement of sustainable biomaterial production due to its high biomass productivity and built-in containment features [14]. Modern sugarcane cultivars are highly polyploid, aneuploid hybrids between *S. officinarum *L. (octoploid, with 2n = 80 chromosomes) and *S. spontaneum *L. (ploidy level of 5-16, with 2n = 40-128 chromosomes). Modern sugarcane cultivars typically have 2n = 100-130 chromosomes, of which approximately 15-20% are derived from *S. spontaneum *and 5% are recombinants derived from both species. Therefore, the genome of modern sugarcane cultivars has at least 10 copies of most homo(eo)logous loci, contributing to the high complexity of its genome [15].

In this study, we used conserved miRNAs to systematically search public EST and GSS databases for sugarcane pri-miRNAs or miRNA precursors. A total of 19 distinct sugarcane pri-miRNAs were identified by our computational protocol, of which nine are monocot-specific. The expression profiles of selected sugarcane miRNAs were monitored by pulsed stem-loop RT-PCR [16] in organs/tissues of a modern cultivar as well as in *S. officinarum *and *S. spontaneum*. To identify target genes of the identified miRNAs, we developed a BLAST-based computational tool to search the NCBI EST and BAC sequences of sugarcane, rice, and sorghum. By using this method, we predicted several target messages, of which one novel target was experimentally tested and confirmed. Finally, we integrated sugarcane miRNA primary precursor and target information into a web-based database (http://sysbiol.cbmeg.unicamp.br/SCmiRNA), which is publicly available. The identification of miRNAs and their targets is important not only to help us learn more about the roles of miRNAs in sugarcane development and physiology, but also to provide a framework for further studies on RNAi-based regulation mechanisms in this highly polyploid species.

## Results and Discussion

### Identification of miRNA primary transcripts in *Saccharum spp*

MiRNAs have been intensively studied in a wide range of plants over the past few years [8], but no systematic and comprehensive study has been performed on sugarcane, one of the most promising biofuel crops worldwide [17]. In order to computationally identify miRNAs in sugarcane, we developed a homology-based strategy based on [7,13] that included the following steps: First, we searched the sugarcane EST and GSS databases to find sequences matching previously known plant miRNAs. Then we predicted the secondary structures of the potential precursor sequences using MFOLD. The third step consisted of an in-house MIRcheck-based script to verify the putative pri-miRNA candidates (parameters described in Methods), followed by a manual inspection to eliminate possible false positives. Finally, closely related EST sequences were blasted against each other to detect redundancy and then further analyzed. We considered as one miRNA precursor those ESTs sharing > 95% identity at the sequence level. This protocol allowed us to retrieve 19 distinct miRNA precursors that were classified into 14 families (Table 1). Amongst them, 18 miRNA precursors were found in the EST database and a single one was found in the GSS sequences, indicating the latter is still a poor source of *in silico *miRNA discovery in sugarcane. Although previous reports have identified some sugarcane miRNA precursors [12,13], in this study we have advanced these findings by systematically analyzing these precursors as well as identifying new ones. For instance, we identified precursors for sugarcane miR827, miR528, miR1128, and miR1432 (Table 1). Moreover, we evaluated the expression patterns of selected miRNAs in different sugarcane tissues/organs (see next section).

**Table 1:** Distinct sugarcane pri-miRNAs identified in this study

miRNA	Sequence source	Sequence ID	Sugarcane *MIR *gene	miRNA Mature Sequence	Location	NM (nt)	LP (nt)	MFEI	Conserved in rice
miR156^a^	EST	TC110664	*SsMIR156b/c*	UGACAGAAGAGAGUGAGCAC	5'	0(0)	411	0.81	Yes
miR159^a^	EST	TC79108	*SsMIR159*	UUUGGAUUGAAGGGAGCUCUG	3'	0(0)	265	0.78	Yes
miR167^a^	EST	TC105794	*SsMIR167*	UGAAGCUGCCAGCAUGAUCUG	5'	0(0)	193	0.93	Yes
miR168^a^	EST	TC97302	*SsMIR168*	UCGCUUGGUGCAGAUCGGGAC	5'	0(0)	104	0.86	Yes
miR169	EST	TC105581	*SsMIR169*	UAGCCAAGGAUGACUUGCCGG	5'	0(1)	148	0.92	Yes
miR396^a^	EST	CA240723	*SsMIR396*	UUCCACAGCUUUCUUGAACUG	5'	0(0)	134	1.02	Yes
miR827	EST	CA215078	*SsMIR827*	UUAGAUGACCAUCAGCAAACA	3'	0(1)	140	1.01	Yes
miR408^a^	EST	TC108481	*SsMIR408a*	CUGCACUGCCUCUUCCCUGGC	3'	0(1)	215	0.67	Yes
	EST	TC74315	*SsMIR408b*	CUGCACUGCCUCUUCCCUGGC	3'	0(1)	283	0.80	Yes
miR437	EST	CA185316^c^	*SsMIR437a*	AAAGUUAGAGAAGUUUGACUU	3'	0	195	1.36	Yes
	EST	CA191146^c^	*SsMIR437b*	AAAGUUAGACAAGUUUGACAU	3'	2	233	0.92	Yes
	EST	CA300436^c^	*SsMIR437c*	AAAGUUAGAGAAGUCUGACUU	3'	1	197	1.36	Yes
miR444	EST	CA186150	*SsMIR444a*	UGCAGUUGUUGCCUCAAGCUU	3'	0	105	1.31	Yes
	EST	CA105916	*SsMIR444b*	UGCAGUUGUUGCCUCAAGCUU/UUGUGGCUUUCUUGCAAGUUG	3'	0	132	1.24	Yes
	EST	TC110432	*SsMIR444c*	UGCAGUUGUUGUCUCAAGCUU/UGUUGUCUCAAGCUUGCUGCC	3'	0	152	1.20	Yes
miR528	EST	CA290495	*SsMIR528*	UGGAAGGGGCAUGCAGAGGAG	5'	0	94	0.86	Yes
miR1128	EST	CA222833^c^	*SsMIR1128*	UACUACUCCCUCCGUCCCAAA	5'	1	275	1.17	No
miR1432	BAC	FJ348731	*SsMIR1432*	CUCAGGAAAGAUGACACCGAC	5'	1	118	1.15	Yes
miR319^b^	EST	TC87836	*SsMIR319*	UUGGACUGAAGGGUGCUCCC	3'	0(0)	n.d.	n.d.	Yes

^a^Sugarcane miRNA families deposited in the miRbase v. 14.

^b^Pri-miRNA identified only by precursor sequence homology. Therefore, we could assign neither LP nor MFEI values to this precursor.

NM, nucleotide mismatches with rice, sorghum and *Arabidopsis *(in parentheses), when applicable.

LP, length of the pre-miRNA.

MFEI, minimal free energy index.

^C^Sugarcane pri-miRNAs with similarity (evalue <e^-10^) to MITE-derived hairpin sequences. miR437 precursors have similarity with *Oryza sativa *MITE-adh type A (2.5e^-17^) while miR1128 precursor is similar to *O. glumipatula *pangrangja MITE element (1.2e^-11^).

The sugarcane miRNA families identified in this study include the six families already deposited in the miRbase (Table 1), indicating the robustness of our approach. Nonetheless, careful inspection of the sugarcane miRNA precursor sequences deposited in the miRbase v.14 and comparison with our analysis revealed some divergences between these databases. For example, the *SsMIR156b/c *(Table 1) was previously annotated as a single stem-loop *MIR156 *precursor (miRbase v.14). However, our analyses revealed that this precursor belongs to a cluster representing a two-tandem microRNA precursor, which is highly similar to its sorghum homolog (90% nt identity) and to the maize *Corngrass1 *microRNA (84% nt identity) [18]. Moreover, genomic DNA PCR amplification from sugarcane hybrid RB 83-5486 using specific primers and subsequent sequencing indicate that *SsMIR156b/c *locus encodes tandem *MIR156 *genes (data not shown). Comparison among the miRbase-derived precursor sequences and with those identified in this study suggests that the 16 previously annotated sugarcane miRNA precursors represent only eight different precursors (Table 1). For instance, we identified only two distinct precursors of miR408, *SsMIR408a *and *SsMIR408b *(Table 1), instead of five (miRbase v.14). Closer inspection suggests that *SsMIR408a *and *SsMIR408b *are likely different alleles of the same locus. This observation is supported by the fact that *MIR408 *genes have been found only as one copy in all plant genomes evaluated to date (miRbase v.14). The discrepancies between our data and previous annotation in the miRbase may be due to the use of SoGI Release 2.2 (July, 2008) that contains substantially more Tentative Consensus (TCs) than the earlier releases, which likely reflect differences in EST clustering or assembling.

In agreement with previous results [7,10], most sugarcane miRNA sequences have uracil as their first nucleotide (13 out of 19 mature sugarcane miRNAs; Table 1). Moreover, sugarcane miRNA precursors displayed high minimal free energy index (MFEI) values (average 1.02 ± 0.22), which is a criterion used for distinguishing miRNA precursors from other types of RNAs. MFEI is a parameter that considers not only the minimal free energy (MFE) value of a particular sequence but also its length and G+C content. MFEI values were calculated as described by [19].

MiRNAs are located either in the 5'-arm or 3'-arm of the stem-loop hairpin pre-miRNA sequences (Table 1). All new identified miRNA precursors could fold into stem-loop structures (see additional file 1), following the rules and parameters reported by [7]. One exception was the EST TC87836, which displays high similarity (e-value 0.0 and 89.5% nt identity) with one of the *MIR319 *precursors present in the draft of the *Sorghum bicolor *genome [20] (see additional file 2). It could not form a suitable stem-loop structure and thus it was not validated by our in-house MIRcheck-based script. This might be due to the fact that the miR319* is located at the 5'end of the sequence, which is not present in the TC87836 sugarcane EST. Nevertheless, based on its extensive homology with sorghum *MIR319 *precursor (see additional file 2), we annotated this TC as a potential *SsMIR319 *precursor (Table 1). That most ESTs do not contain their entire 5'-end sequence information undermines EST databases as sources for miRNA precursor searching. Based on the example given in this study, it may be interesting to develop rules and parameters to assign EST sequences as miRNA precursors based only upon extensive nucleotide identity with precursors from highly closely related species.

Not only *SsMIR319*, but several sugarcane pre-miRNAs show high sequence similarity with their sorghum homologs (values between 86% and 94% nt identity). Sorghum and sugarcane are each other's closest relatives among cultivated crops. They belong to the *Andropogoneae *tribe and diverged from a common ancestor around 8-9 Myr ago [15]. Based on genomic sequence comparisons [15,20], it is likely that sugarcane and sorghum did not have sufficient time to diverge, which reflects the high degree of identity observed between their miRNA precursors.

**Figure 1:**
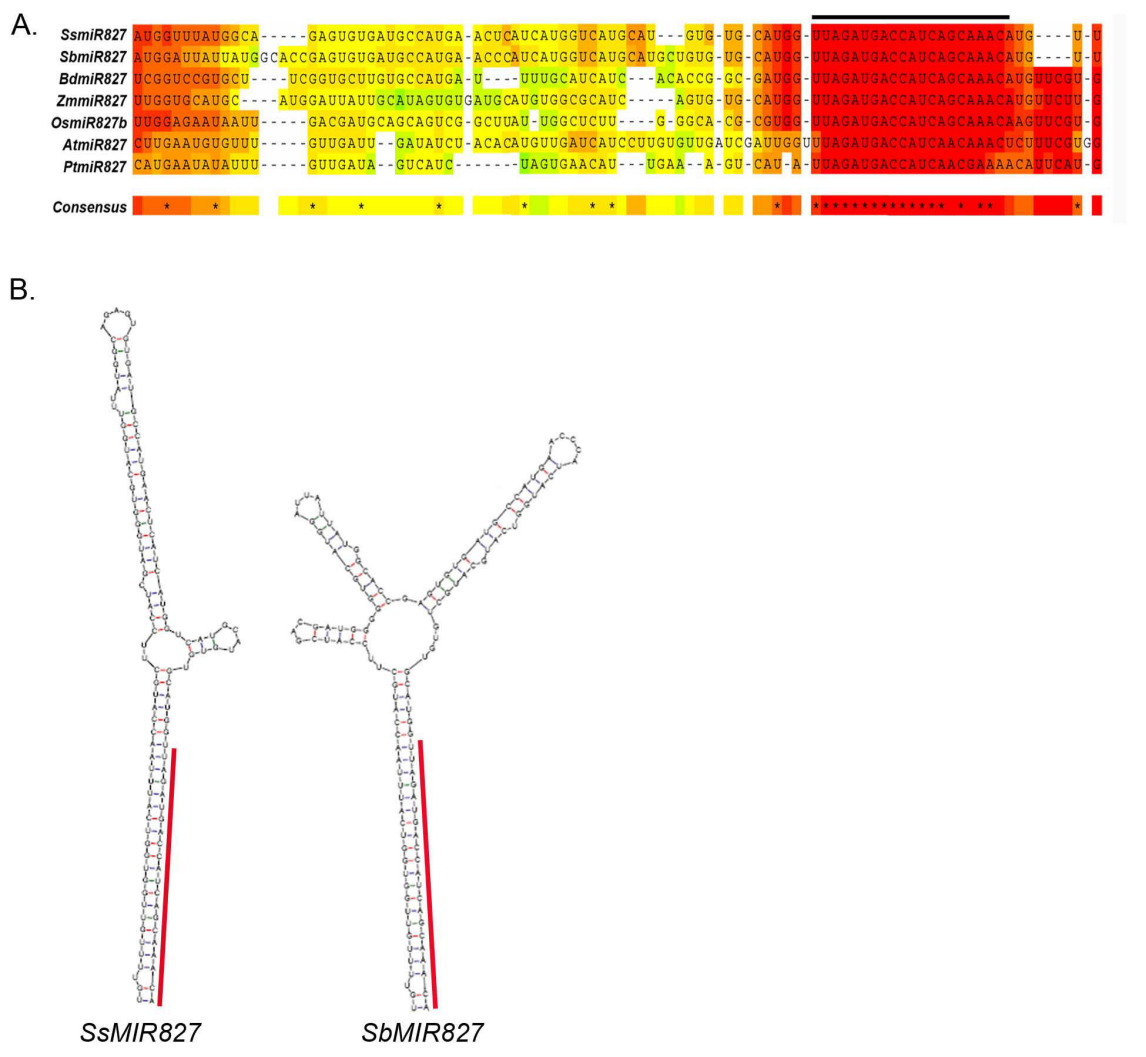
**New sorghum miR827 precursor is highly similar at sequence and secondary structure levels with its sugarcane homolog. **(**A**) Partial multiple sequence alignment of miR827 precursors from *Saccharum spp *(Ss), *Sorghum bicolor *(Sb), *Oryza sativa *(Os), *Zea mays *(Zm), *Brachypodium distachyon *(Bd), *Arabidopsis thaliana *(At), and *Populus trichocarpa *(Pt). Sequence alignment was performed using the T-Coffee program [21]. Black line on top of the alignment indicates the mature miRNA sequence. (**B**) Conserved secondary stem-loop structures of pre-miR827 in sugarcane (Ss) and sorghum (Sb). Red line marks the mature miR827 sequence.

This feature allowed us, by using sugarcane *MIR827 *pre-miRNA sequence, to identify the sorghum *MIR827 *precursor (Figure 1), which was not annotated in previous work [20]. Sorghum *MIR827 *precursor is located at chromosome 4 (position 50273627 to 50273779). The mature miR827 is highly conserved among grasses and displays few mismatches with sequences from *Arabidopsis *and *Populus *(Figure 1A; [21]). The new sorghum miRNA precursor was validated by our in-house MIRcheck-based script and it showed high similarity with its sugarcane homolog not only at the sequence level, but also at a secondary structure level (Figure 1B).

Among the monocot-specific miRNA precursors, we have identified three potential precursors of microRNA444 (Table 1). Interestingly, *SsMIR444b *and *SsMIR444c *contain tandem and overlapping mature miRNA sequences (additional file 1), similar to *MIR444 *precursors identified in rice and sorghum [20,22]. At least in rice, such precursors are able to generate natural antisense miRNAs, or nat-miRNAs. The production of nat-miRNAs depends upon sense/antisense transcription and alternative splicing of the precursors prior to DCL1 cleavage [22]. These nat-miRNAs seem to be restricted to monocot graminae, indicating this new pathway is less than 50 million years old.

**Figure 2:**
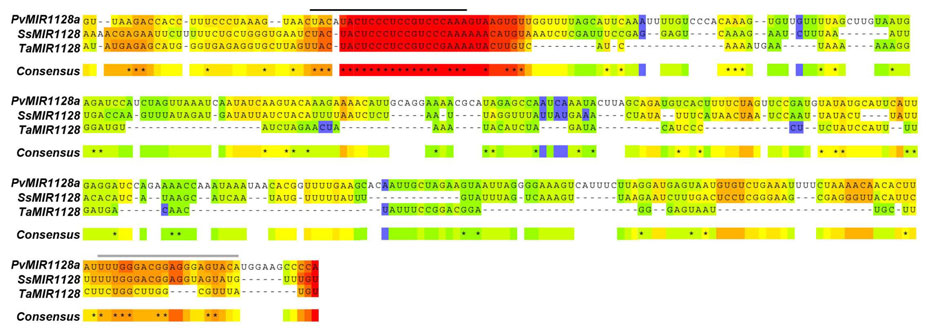
**Partial conservation of mature miR1128 among sugarcane, switchgrass, and wheat. **Multiple sequence alignment of sugarcane pre-miRNA1128 (*SsMIR1128*) with its homologs in switchgrass (Pv) and wheat (Ta). The alignment was done using the T-Coffee program. Black line indicates the mature miR1128 while gray line indicates the miR1128* sequence.

Recent works suggest that some plant and human miRNA families are derived from a subset of DNA-type transposable elements (TEs) called miniature inverted-repeat transposable elements (MITEs; [23,24]). MITEs evolved from corresponding ancestral full-length (autonomous) elements that originally encoded short interfering RNAs (siRNAs). Piriyapongsa and Jordan [24] found several examples in rice and *Arabidopsis *supporting the notion that evolutionary intermediates may exist as TEs that encode both siRNAs and miRNAs. Moreover, Voinnet [8] suggests an association of recently evolved miRNA families with MITEs. Thus, we compared the identified sugarcane pri-miRNA sequences against the Gramineae Repeat database (http://plantrepeats.plantbiology.msu.edu/gramineae.html) using BLASTN (e-value <e^-10^) to identify possible MITE-derived hairpin precursors. Only miR1128 and all three miR437 precursors presented substantial similarity with known MITEs (Table 1). Accordingly, their maize homologs also have similarity with MITE-derived hairpin sequences [25]. It has been shown in *Arabidopsis *that miRNA genes evolved via local inverted duplication events, which generated sequences capable of folding back into hairpin structures when expressed [26]. Similarly, over the course of evolution, MITEs might have stimulated the RNAi biogenesis enzymes to process hairpin-like structures to generate miRNAs with endogenous gene regulatory functions [24]. We were able to detect mature miR1128 by RT-PCR - as shown in the next section - and sequencing of the generated amplicon confirmed its identity (data not shown). Moreover, multiple sequence alignment of pre-miR1128 from sugarcane, switchgrass (*Panicum virgatum*) and wheat (*Triticum aestivum*) [27,28] suggests partial conservation of the miR1128 and miR1128* among these species, but not the surrounding precursor sequences (Figure 2). Along with other requirements [7,8], the conservation of the miRNA and miRNA* sequences in the precursor is a critical parameter to define a miRNA-generating locus. Taken together, our data support the miRNA status of the sugarcane miR1128. However, we cannot rule out the possibility that MITE-associated miRNAs may lose their miRNA status in the future [25].

Given the limited number of sugarcane EST and GSS sequences available as well as existent sequencing errors, the frequency of candidate miRNA precursors identified in this study is comparable to others using such databases [29,30]. It is noteworthy that all miRNAs reported in this study have been identified using previously known miRNAs from several plant species. Therefore, we did not uncover miRNAs that are specific to sugarcane. Further investigations that employ small RNA libraries combined with computational approaches are needed to identify sugarcane-specific miRNAs.

### Expression patterns of sugarcane miRNAs

**Figure 3:**
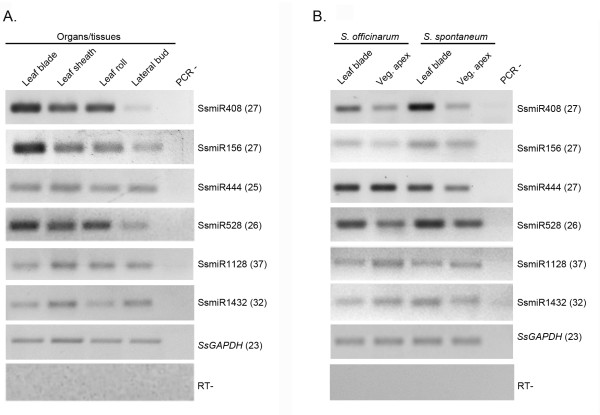
**Expression profiles of miRNAs in sugarcane organs/tissues. **(**A**) Stem-loop pulsed RT-PCR of SsmiRNAs in organs/tissues of sugarcane hybrid RB 83-5486. (**B**) Stem-loop pulsed RT-PCR of SsmiRNAs in organs/tissues of the *S. officinarum *and *S. spontaneum*. *Saccharum spp. GAPDH *(*SsGAPDH*; TC77224) was used as a loading control. RT- (reaction without RT) and PCR- (reaction without cDNA) are shown as negative controls. Numbers in parentheses represent PCR cycles for each amplicon.

The expression pattern of a miRNA in organs/tissues might provide initial clues regarding its biological function. Therefore, we evaluated the expression of selected miRNAs identified in this work (Table 1). We have chosen one miRNA poorly conserved (miR408), one highly conserved among plant species (miR156), and four potential monocot-specific miRNAs (miR444, miR528, miR1128, and miR1432). In this study, stem-loop RT-PCR approach was applied to detect mature miRNA species in distinct organs/tissues from the commercial sugarcane hybrid RB 83-5486. The miRNAs were detected in all organs/tissues analyzed, although with distinct expression profiles (Figure 3A). Transcripts of miR408 accumulate at high levels in all organs/tissues but lateral buds, while miR156 accumulates at higher levels only in leaf blade tissues. Sugarcane miR444 and miR1128 seem to be similarly expressed in the organs/tissues evaluated (Figure 3A). miR1432 mature transcripts accumulate at higher levels in leaf sheath and lateral buds, whereas miR528 transcripts were detected at lower levels in lateral buds. It is noteworthy that all tested SsmiRNAs, though at variable levels, are expressed in lateral buds (Figure 3A). Sugarcane is typically propagated via rhizomes, which contain one or more lateral buds. The new plantlet will arise from these buds and further develop into mature plants (http://sugarcanecrops.com). Therefore, efficient bud outgrowth is an extremely important step for the initial development of sugarcane. It is possible that some of these miRNAs play important roles in the genetic regulation of sugarcane lateral bud outgrowth. Functional studies may provide clues on the possible roles of these miRNAs in the early development of sugarcane.

We also compared the expression profiles of these miRNAs between *S. officinarum *and *S. spontaneum *to evaluate whether both species produce detectable mature miRNA molecules. All miRNAs are detected in the evaluated organs/tissues from these two closely related species (Figure 3B). Although most miRNAs seem to accumulate similarly in both species, some presented variations in abundance when comparing the same organs/tissues at similar developmental stages of *S. officinarum *and *S. spontaneum*. For example, miR444 is slightly more abundant in vegetative apex of *S. officinarum*. In contrast, miR408 accumulates at higher levels in leaf blade tissues of *S. spontaneum *(Figure 3B). Similar data was observed for miRNAs accumulating in some organs/tissues of stable *Arabidopsis *allopolyploids [31]. The relatively low variation in miRNA accumulation between these species is likely a reflection of their level of ploidy. Highly polyploid species might have developed a genetic buffering against extensive miRNA expression variation in particular organs/tissues or developmental stages to maintain target gene expression stability across generations of ploidy [31]. Our data also present the possibility that both ancient species contributed similarly to the miRNA-based regulatory pathways present in modern sugarcane hybrids. It will be interesting to test whether all target loci in hybrid modern cultivars are down-regulated by miRNAs from one ancient progenitor or from both.

**Figure 4:**
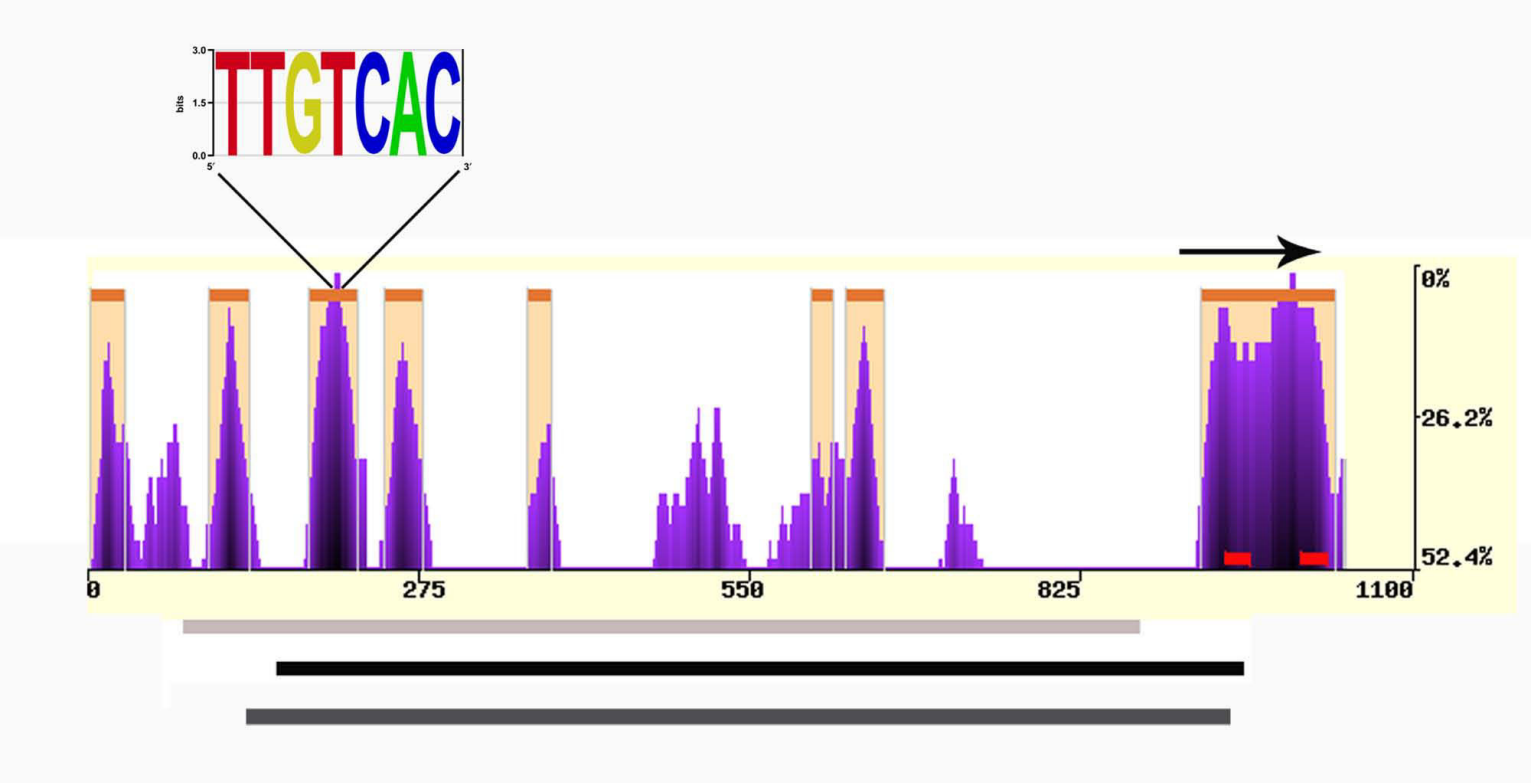
**Conserved elements in the *MIR1432 *locus of sugarcane, sorghum, and maize. **eShadow plot shows the HMMI (Hidden Markov Model Islands) predictions (in beige) of potential evolutionary conserved regions along the *SsMIR1432 *locus compared with its sorghum and maize counterparts. Purple plot bars represent the conserved regions (sliding window size of 35 bp). Red bars in the plot indicate the sugarcane mature miR1432 (left) and miR1432* (right) positions. The arrow on the top of the plot represents the pre-miR1432 position. The x axis represents nucleotide length of the sequence while the y axis represents the percentage of nucleotide variation. Bars underneath the plot mark putative CpG/CpNpG islands present in the *SsMIR1432 *locus (light gray), *SbMIR1432 *locus (black), and *ZmMIR1432 *locus (dark gray). A putative auxin response element (AuxRE) is shown on the top of the plot as an example of a highly conserved TFB site among these three loci.

The final spatiotemporal accumulation of mature small RNAs relies, at least in part, upon the transcriptional control of *MICRORNA *(*MIR*) genes [32] and such regulation may be conserved among closely related species. To gain more insight into the transcriptional regulation of the sugarcane *MIR *genes, we analyzed *in silico *the *SsMIR1432 *locus, which has available genomic sequences (Table 1). Firstly, we employed eShadow software [33] to search for evolutionary conserved regions in *MIR1432 *locus from sugarcane, sorghum, and maize. We detected several potentially conserved regions, of which most are localized upstream of the predicted pre-miRNA and one highly conserved region includes the pre-miR1432 (Figure 4). Secondly, we scanned for putative conserved transcription factor binding (TFB) sites as well as for tandem repeats and CpG/CpNpG islands using JASPAR (http://jaspar.cgb.ki.se) and PlantPAN [34] databases, respectively. CpG/CpNpG islands are regions of the genome typically associated with promoters and 5' ends of several genes. Hypo or hypermethylation of CpG/CpNpG islands in plants are of considerable interest because they relate to patterns of gene regulation, epigenetic phenomena, and chromosome structure [35].

Although we did not detect any tandem repeats, the CpG/CpNpG islands found in the three *MIR1432 *loci overlap broadly with the possibly conserved regions upstream of pre-miR1432. These regions also included common predicted TFB sites for the investigated species, such as an auxin response element (AuxRE) (Figure 4). Taken together, these findings suggest the promoter regions of the sugarcane *MIR1432 *locus share conserved elements with its sorghum and maize homologs. Such elements might be biologically important for the final organ/tissue localization of miR1432 mature species and, consequently, for target down-regulation. It has recently been reported an evolutionary sequencing comparison for the *MIR319a *locus in *Arabidopsis *and related *Brassicaceae*. Reporter experiments have demonstrated that regions under stronger evolutionary constraints contain important information for *MIR319a *transcription [36]. As more sugarcane genomic sequences become available, it will be interesting to verify whether most, if not all homologous miRNAs between sorghum and sugarcane also share conserved elements in their promoters.

### Potential targets of sugarcane miRNAs

Previous studies demonstrated that miRNAs regulate gene expression mainly by binding to perfect or near-perfect complementary sites of mRNA sequences [37-39]. Such behavior indicates that plant miRNA targets can be predicted by simple sequence homology-based searches. Using an in-house BLASTn-based algorithm (described in Methods), we identified a total of 46 potential distinct target sequences for the 14 identified sugarcane miRNA families. Consistent with the essential roles of miRNAs in regulating a variety of biological processes in plants [8], sugarcane target genes seem to be associated not only with development but also with diverse physiological processes (Table 2). Because NCBI sugarcane EST database is limited and its corresponding proteins have not yet been fully annotated, we have additionally applied the same search for rice and sorghum protein-coding sequences. Most sugarcane miRNA targets identified here have homologs in rice and sorghum (Table 2). Although it is unlikely that true targets have been missed in our search, it is important to mention that BLAST-based search strategies have limitations to detect some targets even if a word size of seven is used. One such example of this are the miRNAs miR395b, miR395c, and miR395f targeting *APS1 *(At3g22890) and *APS3 *(At4g14680) genes within *Arabidopsis *[40]. The longest stretch of matching base pairs is six, which falls under the minimum word size employed by BLAST [40].

**Table 2:** Target genes for 14 sugarcane miRNA families

miRNA	**Target gene**^**a**^	Putative function	**Conservation**^**b**^
miR156	TC96571(1), TC76232(1), CF571975(1), CA188863(1), TC98485(1), TC95509(1), TC87521(1)	Squamosa promoter binding protein (SBP)	Yes
miR159	TC94752(4)	Myb protein-like protein	Yes
	CA229394(3), CA168176(3)	Hypothetical protein	No
miR167	CA232593(4), CA292372(4), CA201181(4), CA070734(4), TC109486(4), TC94174(4), TC82328(4), TC76219(4), TC74847(4)	Auxin response factor	Yes
miR168	TC85238(4), CA099906(4), TC85149(4), TC74893(4)	ARGONAUTE1 (AGO1)-like protein	Yes
miR169	TC106305(4), TC103899(4), TC100837(4)	CCAAT-box transcription factor complex	Yes
miR319	TC111376(2)	TCP family transcription factor	Yes
miR396	TC97707(3), TC91031(3)	Growth-regulating factor	Yes
miR408	CA133327(2), TC105472(2), TC82603(2)	Basic blue copper protein-like protein	Yes
miR827	CA186401(0)	SPX domain-containing protein	Yes
miR437	CA112342(0), CA093244(0)	protein kinase-like protein	No
miR444	CA269598(0)	MIKC MADS-box transcription factor	Yes
	CA208272 (2), CA282606 (2)	Hypothetical protein	No
	CA105902 (2), TC90577 (2)	putative nicastrin	No
miR528	TC90826(1), TC99902(1), CA213231(1)	Cu^2+^-binding domain-containing protein	Yes
miR1128	TC97663(2), CA146047(2)	Hypothetical protein	No
miR1432	CA115772(3)	Calcium-transporting ATPase	Yes

^a^ID of potential target sequences. Numbers in parentheses represent mismatches between the miRNA and its target sequence.

^b^Presence or absence of rice homolog genes containing conserved miRNA recognition sites.

**Figure 5:**
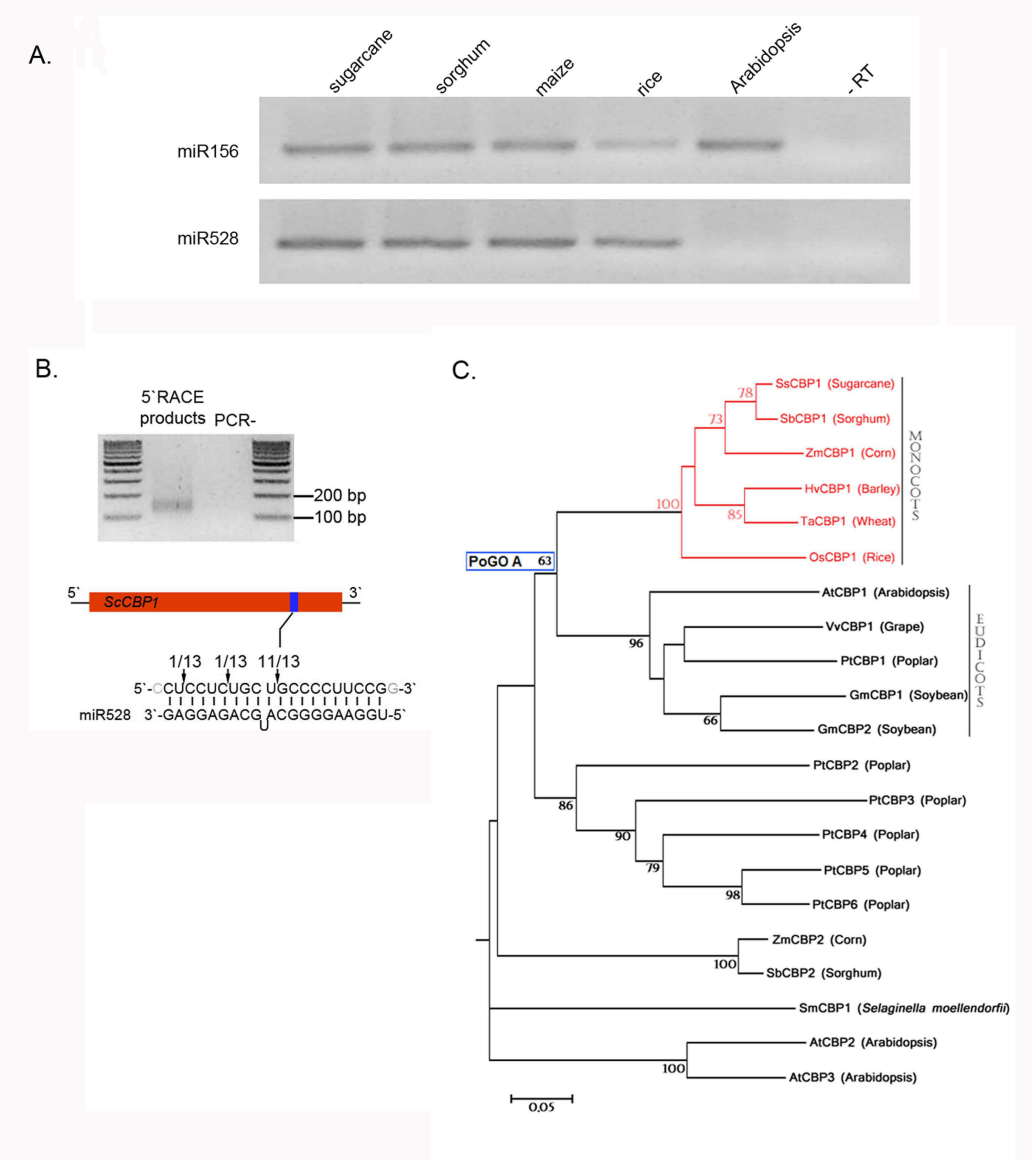
***SsCBP1 *is a target of the monocot-specific miR528. **(**A**) Stem-loop pulsed RT-PCR to detect miR528 transcripts in tissues of sugarcane, sorghum, maize, rice, and *Arabidopsis*. The highly conserved miR156 was used as an internal control. (**B**) Mapping of *SsCBP1 *mRNA cleavage sites by RNA ligase-mediated 5'RACE. Top panel shows the 4% agarose gel containing the expected 160 nt amplicon using specific reverse primers of *SsCBP1 *and RNA ligase-mediated 5'RACE primers. Bottom panel depicts the miR528-complementary site in the target mRNA and the miRNA. Watson-Crick pairing (vertical dashes) is indicated. Arrows indicate the 5' termini of mRNA fragments isolated from sugarcane, as identified by cloned 5'RACE products, with the frequency of clones shown. (**C**) Inferred phylogenetic relationships among SsCBP1 orthologous. p-distances were calculated from amino acid alignments of conserved blocks, and tree topology was inferred with the Neighbor-Joining method. The red branches show the monocot group of CBP genes containing the predict target site for miR528. This monocot group of genes belongs to a possible group of angiosperm orthologs (PoGO A). None of the eudicot sequences within this PoGO contain miR528 predicted target site. The 10 most closely related sequences out of PoGO A were included in this analysis. Only bootstraps values higher than 50% are shown for 1000 replicates. Sequence references are given in Methods.

Interestingly, some target genes that are conserved across angiosperms seem to have lost their miRNA-based regulation in specific lineages [41]. One such example seems to be the new targets for the possible monocot-specific miR528 (Table 2). The three identified ESTs encode Cu^2+^-binding domain-containing proteins (referred to hereafter as SsCBPs; *Saccharum spp. *Cu^2+^-binding domain-containing proteins). To evaluate the relationship between this lineage-specific miRNA and its angiosperm-conserved targets, we initially investigated the accumulation of mature miR528 transcripts in distinct monocots and in the core eudicot *Arabidopsis*. As expected, miR528 transcripts were detected in all graminaceous monocots but not in *Arabidopsis *(Figure 5A). Although targets for the miR528 have been recently predicted in maize [42], no experimental validation has been done to confirm such predictions. Thus, we used the RLM-RACE method to map the cleavage sites in one of the predicted *SsCBPs*(*SsCBP1*, TC90826). As expected, most 5'-ends of the *SsCBP1 *mRNA fragments were mapped to the nucleotide that pairs to the tenth nucleotide of the microRNA, confirming its cleavage guided by miR528 (Figure 5B).

To gain more insight into the evolutionary history of the CBPs, we performed a phylogenetic analysis using SsCBP1 sequence as a query to search for homologous proteins within genomic and EST databanks of a set of green plants, including angiosperms, basal land plants, and green algae (Viridiplantae 1.0; see Methods). Our analysis revealed that SsCBP1 belongs to a Possible Group of Orthologous (PoGO A; for a definition and criteria for PoGO, see [43]) that integrates only angiosperm sequences (Figure 5C). The simplest explanation is that these genes share a common origin within the last common ancestor of angiosperms. Interestingly, the miR528-target recognition site is only present within monocot genes from PoGO A (data not shown). All eudicots orthologous to SsCBP1 from *Arabidopsis*, poplar, grape, and soybean genomes completely lack the miR528-target recognition site, suggesting that miR528 is indeed a monocot-specific microRNA. Taken together, our findings support the notion that the regulation of *SsCBP1 *by miR528 is shared at least within graminaceous monocots, and this miRNA-based post-transcriptional regulation evolved exclusively within the monocots lineage after the divergence from eudicots. Further studies on plant CBPs are needed to define their physiological role(s) and the possible evolutionary advantages given by the miR528-based post-transcriptional regulation of monocot SsCBP1 orthologs.

## Conclusions

Our findings indicate that several sugarcane miRNA precursors share high homology with their sorghum's possible orthologous beyond miRNA mature sequence. In the case of pre-miR1432, which was obtained from genomic sequences, we found precursor-surrounding regions conserved among sugarcane, sorghum, and maize homologs. This finding indicates these genes may share common genetic and epigenetic regulatory programs. However, further work that includes additional homologous sequences from other closely related species is required to confirm such conservation. Our data also indicate that sugarcane miRNAs are expressed in commercial hybrids as well as in the ancient progenitors *S. officinarum *and *S. spontaneum*. Our approach leads to the prediction of several conserved and non-conserved sugarcane miRNA targets in the available EST and genomic databases. The data is available in the public website (http://sysbiol.cbmeg.unicamp.br/SCmiRNA) that will be continuously updated to incorporate future miRBase updates. Our findings will be a useful resource toward tracing the evolution of small RNA-based regulation in sugarcane and related species. Most importantly, this study will serve as a foundation for future research into the functional roles of miRNAs and their target genes in this important biofuel crop.

## Methods

### Plant material and RNA extraction

Leaf blade and sheath tissues were collected from three-week-old sugarcane seedlings (hybrid RB 83-5486) grown in greenhouse conditions. Mature six-month-old plants of the same hybrid were used to obtain lateral buds and leaf roll (apical meristem plus leaf primordia) tissues. We also collected tissues from *Saccharum officinarum *(accession Muntok, Java) and *S. spontaneum *(accession SES205A). Five-month-old plantlets cultivated *in vitro *were transferred to greenhouse conditions. After one month, leaf blade and vegetative apex tissues (pool of four plantlets) were harvested from both species. Tissues were also collected from whole three-week-old seedlings of sugarcane hybrid (RB83-5486) and *Sorghum bicolor *(BTx623), four-week-old seedlings of *Zea mays*, one-month-old plantlets of *Oryza sativa *(ssp. japonica cv Nipponbare), and from one-month-old plantlets of *Arabidopsis thaliana *(Columbia). Total RNA was extracted using Trizol reagent according to manufacturer's instructions.

### Stem-loop reverse transcriptase (RT)-PCR

Stem-loop RT and PCR primers for sugarcane miR408, miR156, miR444, miR528, miR1128, and miR1432 were designed according to [16] (see Additional file 3). Total RNA was treated with DNAse I (Promega) to eliminate any residue of genomic DNA. Six-hundred nanograms of DNAse-treated RNA were used to generate the first strand cDNA [16]. Oligo(dT) primer was added to the reaction for further normalization with the endogenous control gene *glyceraldehyde-3-phosphate dehydrogenase *(GAPDH; [44]). The reaction mixture was placed at GeneAmp9700 thermocycler (Applied Biosystems) and incubated at 16°C for 30 minutes, followed by 60 cycles of pulsed reverse transcription at 30°C for 30 seconds, 42°C for 30 seconds, and 50°C for one second.

cDNA dilutions were used for PCR reactions as following: 1.0 μL of cDNA, 1.5 mM Magnesium Sulfate, 0.25 mM each dNTP, 10 pmol each primer, and 1 U of Taq DNA Polymerase (Fermentas). The reactions were placed in the thermocycler with the following conditions: 94°C for two minutes and appropriate cycle numbers of 94°C for 20 seconds, 60°C for 30 seconds, and 72°C for 45 seconds. All reactions were repeated at least three times.

### Genomic PCR

Prior use of *SsGAPDH *(accession TC77224) as a control to evaluate miRNA accumulation in sugarcane ancient wild species, the efficiency of its primers was tested in genomic DNA from leaves of *S. officinarum *(accession Muntok, Java) and *S. spontaneum *(accession SES205A; see additional file 4). Thirty nanograms of genomic DNA were used as a template for PCR reactions. The reactions were placed in the thermocycler with the following conditions: 94°C for three minutes and 32 cycles of 94°C for 20 seconds, 58°C for 30 seconds, and 72°C for 45 seconds. The reactions were repeated twice.

### Analysis of 5'RACE

Five micrograms of total RNA from sugarcane plantlets (hybrid RB 83-5486) were ligated to a RNA adapter, in a reaction mixture containing 0.5 U/μL of T4 RNA Ligase, 4 U/μL RNAse inhibitor, and 1 mM ATP. The subsequent steps were performed according to the manufacturer's guide of the GeneRacer kit (Invitrogen). The first PCR was done using the following *SsCBP1 *specific primer: 5'-GAAAGCCCTCTCCGCCAGC. The PCR reaction was subsequently used as a template for a semi-NESTED PCR with an internal *SsCBP1 *specific primer (5'-GCGCCGTCGCCGCACCC). After amplification, 5'RACE products were gel-purified and cloned, and at least 13 independent clones were randomly chosen and sequenced.

### Sugarcane miRNA precursor identification

Sugarcane ESTs and GSSs were retrieved from The Gene Index Program (116,588 unique sequences; Release 2.2, July 2008) and NCBI, respectively. The sequences were used as drivers for a BLASTX search (e-value e^-10^) against the NCBI protein sequence database (September 2008). All potential no hit sequences were recorded as a distinct dataset. Recorded miRNAs from plants were obtained from the miRBase (over 2,300 miRNA sequences; Release 14.0) [45] and used as drivers for BLASTN search of sugarcane miRNA precursors in the aforementioned dataset, similarly as described by Zhang *et al.*[13]. We allowed 0-3 nt mismatches or gaps between drivers and database sequences. The BLASTN parameters were adjusted to expected values of 1000 and number of descriptions and alignments of 1000. The default word-match size between the query and the database sequences was seven with a low complexity filtering ability. We also employed BLAST searches to remove sugarcane sequences similar to tRNAs, ncRNAs (http://biobases.ibch.poznan.pl/ncRNA), snoRNAs (http://bioinf.scri.sari.ac.uk/cgi-bin/plant_snorna/home) or other RNAs found in the Rfam database [46].

Wherever available, precursor sequences of approximately 620 nt were extracted (300 nt upstream of and 300 nt downstream from the BLAST hits) and used for hairpin structure predictions using MFOLD3.2 algorithm [47]. Number of structures, free energy, miRNA-like helicity, number of arms per structure, size of helices within arms, and size/symmetry of internal loops within arms were analyzed by our in-house MIRcheck-based script [37], following manual inspection. RNA sequences were considered miRNA precursor candidates only if they fitted the following criteria: (1) the RNA sequence could fold into an appropriate stem-loop hairpin secondary structure; (2) mature miRNA site was located in one arm of the hairpin structure; (3) the mature miRNA sequence was located in the same arm of the hairpin as its homolog in other plant species; (4) mature miRNA had six or fewer, and one or more, mismatches with the miRNA* sequence in the opposite arm; (5) no break in miRNA* sequences; (6) predicted secondary structures had MFEI values higher than 0.65 [12,19], negative MFEs, and 30-70% G + C contents; (7) two base pairs of maximum consecutive mismatches between miRNA and miRNA*; (8) a minimum of two bases pairing after the alignment between the predicted miRNA sequence and its opposite miRNA* sequence within the secondary structure; (9) and a final stem loop with a minimum of 60 nt.

### Predicting sugarcane miRNA targets

Sugarcane miRNA mature sequences were used to BLAST search for possible gene targets present in the SoGI database and available BAC sequences. To minimize the number of false positives, 21-nt miRNA sequences were initially divided into three blocks of eight (block 1), three (block 2), and 10 bp (block 3). The maximum mismatches permitted in each block for the mRNA:miRNA duplex were two, zero, and three, respectively. To more thoroughly assess the mRNA::miRNA potential pairing, we additionally developed a more sensitive computational approach to identify target candidates. Each miRNA complementary site was scored, with perfect matches given a score of zero. Points were added for each G:U bulge (0.5), non-G:U mismatch (one), and bulged nucleotide in the miRNA or target strand (1.5). Only SoGI/BAC sequences that scored ≤3.5 points were further considered as potential miRNA targets. Closely related sequences were blasted against each other and analyzed. Sequences sharing ≥95% of identity at nucleotide level were considered as one gene target.

### SsCBP1 comparative sequence analysis

Comparative analysis of sugarcane SsCBP1's possible orthologous in green plants was done by constructing a phylogenetic tree containing highly similar plant sequences. A BLASTX search was performed using *SsCBP1 *as query against a green plant protein dataset of 365,187 protein sequences obtained from several completed genomes (*Arabidopsis thaliana*, version 7.0 - http://www.arabidopsis.org* ;Populus trichocarpa*, version 1.1 - http://genome.jgi-psf.org/Poptr1_1/Poptr1_1.home.html; *Glycine max*, version 0.1 - http://www.phytozome.net/soybean.php* ;Oryza sativa*, version 5.0 - http://www.tigr.org/tdb/e2k1/osa1/pseudomolecules/info.shtml* ;Sorghum bicolor*, version 1.4 - http://genome.jgi-psf.org/Sorbi1/Sorbi1.home.html; *Selaginella moellendorffii*, version 1.0 - http://genome.jgi-psf.org/Selmo1/Selmo1.home.html; *Physcomitrella patens patens*, version 1.1 - http://genome.jgi-psf.org/Phypa1_1/Phypa1_1.home.html; *Volvox carteri*, version 1.0 - http://genome.jgi-psf.org/Volca1/Volca1.home.html; *Chlamydomonas reinhardtii*, version 3.0 - http://genome.jgi-psf.org/chlre3/chlre3.home.html; *Ostreococcus lucimarinus*, version 2.0 - http://genome.jgi-psf.org/Ostta4/Ostta4.home.html; *Ostreococcus tauri*, version 2.0 - http://genome.jgi-psf.org/Ostta4/Ostta4.home.html; *Micromonas pusilla *CCMP1545, version 2.0 - http://genome.jgi-psf.org/MicpuC2/MicpuC2.home.html; *Micromonas *strain RCC299, version 2.0 - http://genome.jgi-psf.org/MicpuN2/MicpuN2.home.html). The conserved domains found among protein sequences were aligned using ClustalW [48] to produce ungapped alignments. The phylogenetic relationship of these aligned sequences was then constructed using the Neighbor-Joining method. Phylogenetic analysis was conducted in MEGA4 software [49]. This process allowed identifying the most probable orthologous sequences of the SsCBP1. EST sequences from barley, wheat, and sugarcane were obtained from "TIGR Plant Transcript Assemblies Database" [50], and cDNA sequences from maize were obtained from MAGI (http://magi.plantgenomics.iastate.edu/). The accession numbers of genes shown in Figure 5 are as follows: SsCBP1 - TC67256; SbCBP1 - Sb02g036870; ZmCBP1 - MAGIv4.0 54669; HvCBP1 - BI947163; TaCBP1 - CK217219; OsCBP1 - Os07g38290; AtCBP1 - At5g26330; VvCBP1 - Sim4.aln-TCVV023209; PtCBP1 - 821987; GmCBP1 - Gm0010 × 00014; GmCBP2 - Gm0133 × 00019; PtCBP2 - 415490; PtCBP3 - 195948; PtCBP4 - 410618; PtCBP5 - 561943; PtCBP6 - 173259; ZmCBP2 - MAGIv4.0 158060; SbCBP2 - Sb01g004320; SmCBP1 - 27471; AtCBP2 - At2g26720; AtCBP3 - At2g31050.

## Authors' contributions

ASZ and RV carried out the molecular biology studies and the bioinformatic analyzes; FAOM and MJS participated in the molecular biology studies and helped analyzing the data; FTSN, LEVB and MV carried out the phylogenetic analyzes; FTSN designed and coordinated the study, and wrote the manuscript. All authors read and approved the final manuscript.

## Supplementary Material

Additional file 1 **supplementary PDF figure1**. Click here for file

Additional file 2 **supplementary PDF figure2**. Click here for file

Additional file 3 **supplementary PDF Table1**. Click here for file

Additional file 4 **supplementary PDF figure3**. Click here for file
