# Supplementary material for: Identification and expression analysis of microRNAs and targets in the biofuel crop sugarcane
Source: BMC Plant Biol. 2010 Nov 24;10:260. doi: 10.1186/1471-2229-10-260 (PMC3017846; doi:10.1186/1471-2229-10-260)
Supplement: Additional file 1 — supplementary PDF figure1. [file 1471-2229-10-260-S1.pdf]

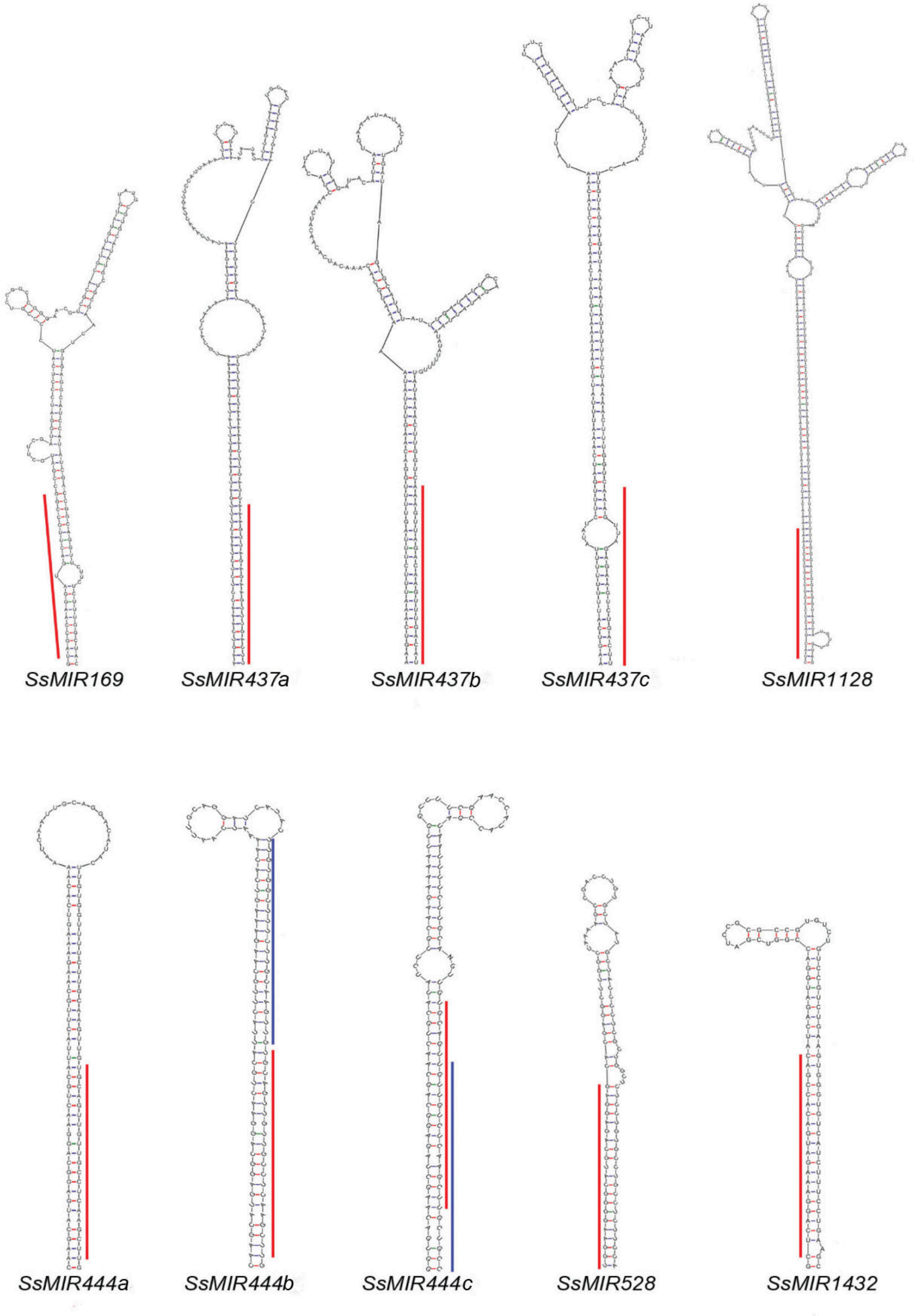

Figure S1. **Pre-miRNA secondary stem–loop structures of newly identified sugarcane miRNAs**.Colored (red and blue) lines indicate the mature miRNA sequences. Secondary structures are predicted using MFOLD3.2 algorithm.
