# Supplementary material for: Identification and expression analysis of microRNAs and targets in the biofuel crop sugarcane
Source: BMC Plant Biol. 2010 Nov 24;10:260. doi: 10.1186/1471-2229-10-260 (PMC3017846; doi:10.1186/1471-2229-10-260)
Supplement: Additional file 2 — supplementary PDF figure2. [file 1471-2229-10-260-S2.pdf]

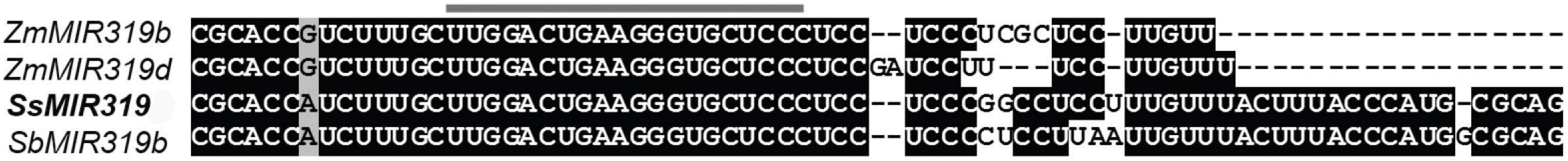


**Figure S2. Conserved sugarcane pre-MIR319 derived from EST sequences**. Multiple sequence alignment of sugarcane pre-MIR319 (SsMIR319) with its homologs in sorghum (Sb) and maize (Zm). Gray line indicates mature miR319 sequence.
