# Supplementary material for: Identification and expression analysis of microRNAs and targets in the biofuel crop sugarcane
Source: BMC Plant Biol. 2010 Nov 24;10:260. doi: 10.1186/1471-2229-10-260 (PMC3017846; doi:10.1186/1471-2229-10-260)
Supplement: Additional file 3 — supplementary PDF Table1. [file 1471-2229-10-260-S3.pdf]

Table S1. Primers used in this study to detect mature sugarcane miRNAs and *GAPDH*

Genes	Stem–loop RT primer (5`-> 3`)	Foward primer (5`-> 3`)	Reverse primer (5`-> 3`)
miR156	GTCGTATCCAGTGCAGGGTCCGAGGTATTC GCACTGGATACGACGTGCTC	GCGGCGGTGACAGAAGAGAGT	GTGCAGGGTCCGAGGT
miR408	GTCGTATCCAGTGCAGGGTCCGAGGTATTC GCACTGGATACGACGCCAGG	GCGGCGGCTGCACTGCCTCTTC	GTGCAGGGTCCGAGGT
miR444	GTCGTATCCAGTGCAGGGTCCGAGGTATTC GCACTGGATACGACAAGCTT	ACTGATGTGCAGTTGTTGYCTC	GTGCAGGGTCCGAGGT
miR528	GTCGTATCCAGTGCAGGGTCCGAGGTATTC GCACTGGATACGACCTCCTC	GTGGTTGGAAGGGGCATGCA	GTGCAGGGTCCGAGGT
miR1128	GTCGTATCCAGTGCAGGGTCCGAGGTATTC GCACTGGATACGACTTTGGG	ACTGATGUACTACTCCCTCCGT	GTGCAGGGTCCGAGGT
miR1432	GTCGTATCCAGTGCAGGGTCCGAGGTATTC GCACTGGATACGACGTCGGT	TCGCGCTCAGGAAAGATGAC	GTGCAGGGTCCGAGGT
GAPDH	-	TTTGAATGGCAAGCTCACTG	GGTGGAAACCAAATCCTCCT
