# Supplementary material for: Identification and expression analysis of microRNAs and targets in the biofuel crop sugarcane
Source: BMC Plant Biol. 2010 Nov 24;10:260. doi: 10.1186/1471-2229-10-260 (PMC3017846; doi:10.1186/1471-2229-10-260)
Supplement: Additional file 4 — supplementary PDF figure3. [file 1471-2229-10-260-S4.pdf]

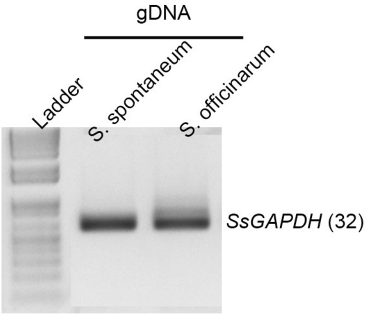

Figure S3. Genomic PCR of *SsGAPDH* (accession TC77224) in *S. spontaneum* and *S. officinarum*.
The efficiency of the *SsGAPDH* primers was evaluated through gDNA PCR in sugarcane ancient wild species. *SsGAPDH* amplicons were similarly generated in both species.The number in parentheses represents PCR cycles.
